# Design of Prefabricated Concrete-Filled Steel Pipe Columns for Pile Beam Arch Subway Stations Based on Carbon Emission Optimization

**DOI:** 10.3390/ma18163854

**Published:** 2025-08-17

**Authors:** Aizhong Luo, Yuting Wu, Tao Li, Xingyu Yang, Yao Liu, Jiajun Shu

**Affiliations:** 1 School of Civil Engineering, Guizhou University of Engineering Science, Bijie 551700, China; aizhongluo@126.com; 2 School of Mechanics and Civil Engineering, China University of Mining and Technology-Beijing, Beijing 100083, China

**Keywords:** PBA method, prefabricated concrete-filled steel pipe columns, carbon emission, design optimization

## Abstract

With the rapid expansion of underground rail transit construction in China, the high carbon emissions associated with subway tunnels and stations have become an increasing concern. This study systematically examines the carbon emissions of prefabricated concrete–filled steel pipe columns (PCSPCs) during the construction phase of a Beijing subway station built via the pile beam arch (PBA) method, applying the life cycle assessment (LCA) methodology as a case study. An analytical framework for the synergistic optimization of carbon emissions and costs was developed. By systematically adjusting key design parameters—such as the column diameter, wall thickness, and concrete strength—it was possible to minimize both carbon emissions and project costs while meeting the ultimate load-bearing capacity requirements. The results indicate that the production phase of PCSPCs accounts for as much as 98.845% of total carbon emissions, with labor, materials, and machinery contributing 10.342%, 88.724%, and 0.934%, respectively. A sensitivity analysis revealed that steel plates have the greatest impact on carbon emissions, followed by steel reinforcement, whereas concrete and cement exhibit relatively lower sensitivities. The ultimate load-bearing capacity of PCSPCs increases with larger column diameters, thicker walls, and higher concrete strength grades, with the relationships displaying a nonlinear trend. The damage modes and performance of PCSPCs under different design parameters were further verified through finite element analysis. On the basis of the optimization algorithm used to adjust the design parameters, the carbon emissions and costs of the PCSPCs were reduced by 10.32% and 21.55%, respectively, while still meeting the load-bearing capacity requirements.

## 1. Introduction

With the continuous rise in global energy demand, greenhouse gas emissions have increased significantly, with carbon emissions identified as the primary driver of global climate change [1,2]. Since 2008, China has been the world’s largest emitter of greenhouse gases and is expected to remain so for the foreseeable future, underscoring its dual role in global climate governance as both a major source of emissions and a key contributor to mitigation efforts [3]. In response, the Chinese government has introduced the “dual carbon” strategic target—achieving peak carbon emissions by 2030 and achieving carbon neutrality by 2060 [4,5]. The transportation sector plays a pivotal role in China’s overall carbon emissions profile, with data showing that, in 2008, carbon dioxide emissions from this sector accounted for approximately 15.9% of the nation’s total end-use carbon emissions. With ongoing urbanization, rapid growth in motor vehicle ownership, and increasing transportation demand, this share is projected to approach one-third by 2030 if effective control measures are not implemented, making rail transit–related carbon emissions one of the primary sources of greenhouse gases. Currently, the total length of urban rail transit lines nationwide has reached 10,566.55 km, with 57 cities having complete and operational systems, of which metro lines account for 8112.23 km—76.77% of the total. As rail transit construction continues to expand, stations, as critical nodes, are subject to increasingly stringent requirements for structural design, engineering safety, and operational efficiency. Among the various design approaches, multispan stations have been widely adopted for their superior space utilization and structural stability, and, within this context, the pile beam arch (PBA) method has become the mainstream construction technique for multispan station development [6,7].

Against the backdrop of advancing a low-carbon transportation system, increasing attention is being given to carbon emissions across the entire life cycle of rail transit. Among these stages, research on carbon emissions during the operational phase is relatively comprehensive, with existing studies primarily examining the effects of factors such as energy consumption by traction power systems, electricity use during vehicle operation, and passenger density on carbon output [8,9]. In contrast, although the construction phase is playing an increasingly significant role in the total carbon emissions profile, systematic research in this area remains insufficient. The construction process involves multiple stages, including the production and transportation of construction materials, fuel consumption by machinery and equipment, and the disposal of construction waste—all of which contribute substantially to carbon emissions [10,11,12,13]. As the proportion of emissions from the construction phase continues to rise within the life cycle emission structure of urban rail transit systems, reducing carbon emissions during this stage has become an increasingly pressing issue. Carbon management in the construction phase is influenced by a range of complex factors, including design schemes, construction processes, and material selection [10,14,15], making it a critical area that must not be overlooked in achieving the low-carbon goals of rail transit development [16,17,18,19]. Prefabricated concrete-filled steel pipe columns (PCSPCs) are key components in the structural systems of PBA stations. These components not only offer high strength and ductility, allowing them to accommodate complex structural design requirements, but also possess excellent seismic performance, making them widely used in engineering projects in earthquake-prone areas [20]. Recent advances, such as Rad’s introduction of residual internal force complementary strain energy constraints within plastic limit analysis, provide promising directions for optimizing pile foundation design under uncertainty [21]. Furthermore, as PCSPCs are predominantly prefabricated in factories, standardized production can be achieved under strict quality control, improving component consistency and reliability. More importantly, this highly prefabricated construction approach reduces energy consumption and construction waste emissions during the building process, offering significant advantages in controlling the carbon footprint [22,23,24]. However, systematic quantitative assessments of carbon emissions from PCSPCs in metro stations constructed via the PBA method are still lacking [25,26,27,28,29].

To address the limitations of the above studies, we propose a strategy to quantify the carbon emissions of PCSPCs at stations on the basis of the PBA methodology and reduce these emissions by optimizing design and construction methods. The life cycle assessment (LCA) approach was applied to systematically define the system boundary for the carbon emissions of PCSPCs at a Beijing PBA station, and a corresponding carbon emission estimation model was developed. By combining the bill of quantities with onsite data, we accurately calculate the carbon emissions during the construction phase, comprehensively quantifying the emissions from labor, materials, and mechanical equipment involved in production, transportation, construction, and installation. We also analyzed the economic costs of PCSPCs from three main perspectives—labor, materials, and machinery—and compared the cost-to-carbon emission ratios to reveal their interrelationship. To further enhance carbon emission performance, a sensitivity analysis was conducted to identify the degree of influence of each factor, and targeted measures for emission reduction were proposed. In addition, a systematic analysis of the ultimate load-bearing capacity of PCSPCs was performed, considering factors such as the concrete strength, geometric parameters, and slenderness ratio. Finite element modeling was then employed to simulate and analyze the mechanical response and failure modes, with the simulation results compared against the calculated values to verify their reliability. Finally, to achieve the dual objectives of minimizing carbon emissions and costs in steel pipe column design, we develop an optimization model based on a multiobjective iterative search algorithm implemented on a numerical calculation platform. This model combines exhaustive parameter grid search with a heuristic single-objective iterative strategy to optimize design parameters such as diameter, wall thickness, and concrete strength under the constraint of meeting ultimate load-bearing capacity requirements to determine the optimal parameter combination. At each iteration, the constraint is evaluated in real time via the ultimate load-bearing capacity formula to exclude infeasible solutions, thereby identifying the minimum-carbon-emission and minimum-cost solutions that satisfy structural safety conditions. This optimization process not only targets emission reduction but also ensures cost-effectiveness, and its results are further validated through finite element analysis, providing both a theoretical basis and practical guidance for achieving more energy-efficient and low-carbon structural designs during the design stage.

## 2. Carbon Emission Calculation Model Based on LCA Methods

### 2.1. Engineering Example

**Figure 1:**
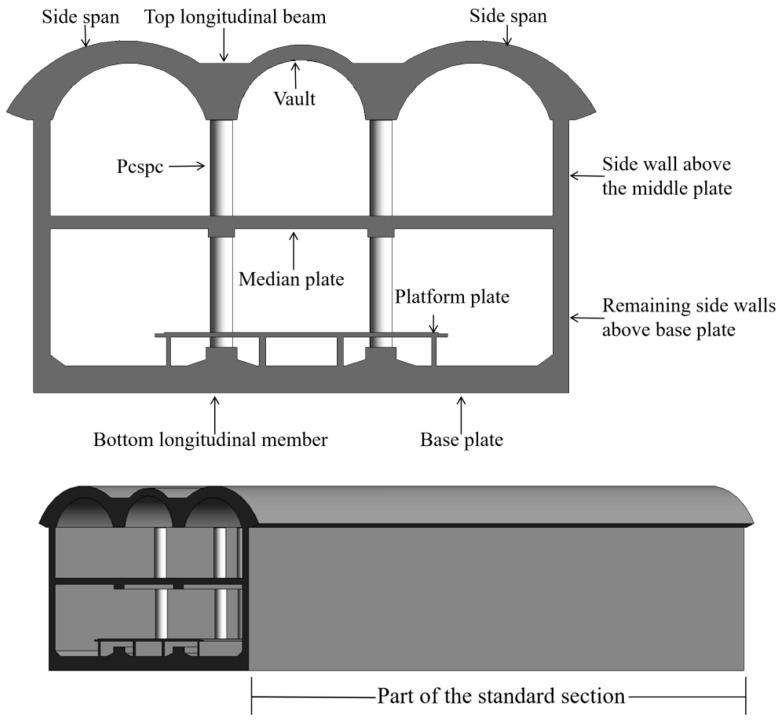
BIM 3D model of a standard section of a station.

On the basis of the life cycle assessment (LCA) framework, a carbon footprint assessment model was developed for PCSPCs during the construction phase of metro stations via the PBA method, with the goal of systematically identifying carbon emission sources and quantifying key influencing factors at each stage. Considering both the carbon reduction and cost control objectives, an optimal construction strategy was proposed. In the engineering case study, the station is an underground island station with a platform width of 16 m, a total length of 279 m, and a standard segment width of 25.2 m. The underground structure adopts a double-layer, double-column, three-span design constructed via the underground cut-and-cover PBA method. The total thickness of the double-layer structural section is approximately 10.8 m, whereas the thickness of the local triple-layer section is approximately 6.6 m. The base slab is located at a depth of approximately 27.2 m. The standard sections of the main station structure were modeled in 3D via BIM technology, as shown in Figure 1. The PCSPCs serve as the core structural elements of the station system and mainly comprise the following components: column steel pipes, inner lining pipes, ring-shaped steel plates at the base of the bottom longitudinal beams, ring-shaped and tension-resistant steel plates at the middle longitudinal beams, steel plates at the top longitudinal beam column caps, flange plates at steel pipe connections, a continuous internal reinforcement cage, and C50 microexpanding concrete poured into the columns.

As a key data source and process support tool for LCA and optimization analysis, BIM serves two main functions: material list generation and component parameter extraction. On the basis of the geometric attributes and material information of component families within the BIM model, the system automatically extracts key data such as structural dimensions, component quantities, steel usage, and concrete volumes for steel pipe columns. These data are standardized and used as inputs for the carbon emission factor database, enabling the construction of carbon emission inventories for the construction phase and the execution of LCA calculations. Moreover, this information supports subsequent cross-sectional optimization of components and cost–carbon trade-off analyses. For construction process visualization and high-carbon process identification, BIM models are used to simulate construction and installation sequences, track changes in component locations, and map construction site layouts. Combined with the carbon emission inventory results, components and processes with high carbon emission intensities are visually highlighted within a 3D interface, helping decision-makers pinpoint key areas for carbon reduction and providing spatial and semantic support for optimizing both design and construction plans.

### 2.2. Scope of Carbon Emission Quantification

The life cycle of an urban metro system can generally be divided into six stages: design, production, transportation, construction, operation and maintenance, and demolition. Given the long-term and lagging nature of carbon emissions during the operational and demolition phases, their environmental impacts are difficult to assess accurately in the short term; therefore, this study does not include carbon footprint analysis for these two phases [30]. The analysis was conducted under two main assumptions: first, although various types of greenhouse gases exist, only CO_2_ emissions were considered, as CO_2_ accounts for the majority of carbon emissions during the construction phase; second, all research data were sourced from actual construction sites, with material usage and energy consumption obtained through onsite measurements to ensure analytical accuracy and reliability. Notably, the LCA method adopted here is limited to the construction phase and does not yet cover the operation or end-of-life stages for two primary reasons: (1) data availability limitations—since this study focuses on the low-carbon optimization of steel pipe column components during subway station construction, information on operational energy use, maintenance cycles, material replacement frequencies, and demolition methods is currently lacking systematic statistical data and standardized modeling criteria, making it difficult to build a reliable quantification model for postconstruction phases and (2) the focus on design flexibility at the construction stage—the construction phase not only presents one of the highest carbon emission intensities in infrastructure projects but also offers the greatest scope for control and adjustment of designs, processes, and material choices, making it the most suitable target for low-carbon design optimization. For these reasons, the system boundary of this study is set to the construction period, with the aim of providing actionable recommendations for carbon reduction strategies during the early design stage.

Second, this study relies on data obtained directly from actual construction sites, with the case data reflecting the real construction process; material quantities and consumption were measured onsite, ensuring the accuracy and authenticity of the analysis. Finally, since this study focuses on prefabricated steel pipe columns and does not involve transportation of construction waste or excavated soil during the transportation phase, carbon emissions from these sources were excluded from the calculation. The system boundaries for this study encompass the production of building materials, transportation of materials and components, and onsite construction and installation. The carbon footprint calculation follows guidelines established by international organizations and research institutions, such as the World Resources Institute. Taking the IPCC Guidelines for National Greenhouse Gas Inventories as an example, the carbon footprint is calculated as shown in Equation (1).

(1) Carbon footprint=Activity level × Carbon footprint coefficient

where activity level, also known as direct data, refers to the time consumed by the construction unit during the construction process, including the time spent by machinery and personnel in the work area. Carbon footprint coefficients are indirect data, referring to the carbon emission coefficients of machinery and equipment, personnel, and materials. Therefore, we limit the scope of carbon emissions during the construction phase of PCSPCs for metro stations on the basis of the PBA method to materials, machinery, and labor.

### 2.3. Quantitative Modeling of Carbon Emissions

The total carbon emissions generated during the construction phase of PCSPCs for metro stations via the PBA method include three parts, namely, the amount of carbon dioxide produced by construction materials *C_m_*, the amount of carbon dioxide produced during transportation *C_t_*, and the amount of carbon emissions generated by the fuel consumption of construction machinery, and the carbon emissions generated manually during the construction and installation phases *C_j_*. The specific calculation process is shown in Equation (2).

(2) $$C=C_{m}+C_{t}+C_{j}$$

On the basis of Beijing’s metro station construction quotas, bill quantities, and onsite survey data, a quantitative analysis of carbon emissions was conducted. Typically, a certain material loss rate is considered in carbon emission calculations to account for potential losses during the process from raw material production to the utilization of building components. However, since the material usage data in this study are derived from actual consumption records at the construction site, they fully reflect the real usage conditions. Therefore, the loss factor is not separately included in the subsequent calculations. The method for calculating carbon emissions is presented in Equation (3).

(3) $$C_{m}=\sum_{i=1}^{n}q_{mi}\times f_{mi}$$

where *q_mi_* is the number of construction materials *i*(*t*), and *f_mi_* is the greenhouse gas intensity factor of the construction materials, *i* = 1, 2, 3, …, *n*, which represents the types of materials considered.

Since carbon emission factors vary over time and by location, a range of factors—including those specific to different provincial regions—were considered [31,32,33], and the most relevant carbon emission factors were selected. The compiled carbon emission factors for the materials are listed in Table 1.

During the transportation phase, the calculation of carbon emissions needs to consider the different loads, types of transport vehicles and energy fuels used. Depending on the specific requirements and actual situation of the construction project, the carbon emissions generated during transportation can be calculated via Equation (4).

(4) $$C_{t}=\sum_{i=1}^{n}q_{ti}\times f_{ti}\times l_{ti}$$

where *q_ti_* represents the quantity of materials (t), *f_ti_* represents the carbon emission factor per unit weight per unit transport distance (tCO_2_/t·km), and *l_ti_* represents the distance between transport material *i* and the construction site (km).

The construction and installation stage includes the loading and unloading stage, and its specific carbon emissions are mainly composed of carbon emissions generated by labor and construction machinery, as shown in Equation (5).

(5) $$C_{j}=m\times f_{jr}+\sum q_{jr}\times f_{mach,J}$$

where *m* represents the number of working days of workers; *f_jr_* represents the artificial carbon emission coefficient; *q_jr_* represents the shift in construction machinery; and *f_mach,j_* represents the greenhouse gas emissions of a unit shift in construction machinery *i*, which can be described as the sum of greenhouse gas emissions of fuel and energy consumed by a machine shift (which refers to the standardized operation of construction machinery for 8 h).

(6) $$f_{mach,J}=\sum r_{fi}\times f_{fi}$$

where *r_fi_* is the fuel consumed in a shift. *f_fi_* is the greenhouse gas emission coefficient of unit consumption of fuel *i*. Similarly, the national quota stipulates the average consumption of each fossil fuel of a machine in a shift.

## 3. Carbon Emission Calculation Results and Analysis

### 3.1. Estimation of Total Carbon Emissions of PCSPCs

During construction, heavy-duty gasoline trucks with a load capacity of 18 tons were used as the primary means of transportation. The carbon emissions generated during the transportation phase, along with the total carbon emissions from PCSPCS components during the construction phase, are presented in Table 2 and Table 3.

**Figure 2:**
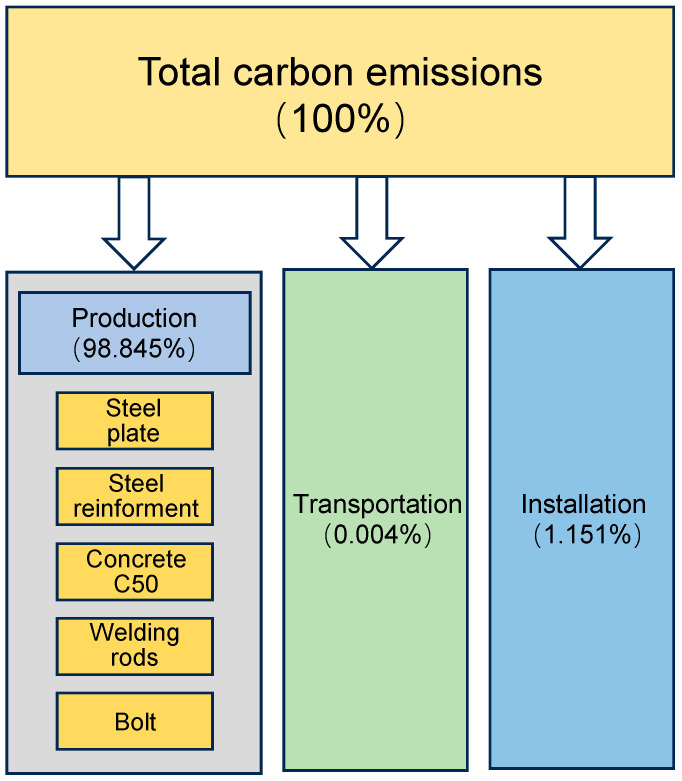
Percentage of carbon emissions at different stages of materialization.

As shown in Figure 2, carbon emissions from the material production stage account for 98.8% of the total carbon emissions during the construction phase, whereas transportation and construction together contribute less than 1.2%. Jeong et al. [34] reported that precast columns and cast-in-place columns account for 98.9% and 95.4% of carbon emissions during the material production stage, respectively. This finding further confirms the dominant role of material production in the overall carbon emissions of the construction process.

According to the above calculations and analysis, carbon emissions during the production phase account for more than 90% of the total emissions. This is attributed to the extensive use of high-energy-consuming building materials such as concrete, steel bars, and steel plates in the construction of PCSPCs. Therefore, it is essential to conduct further analysis on materials—particularly concrete—that are widely used in PCSPCs to carry out targeted emission reduction research.

**Figure 3:**
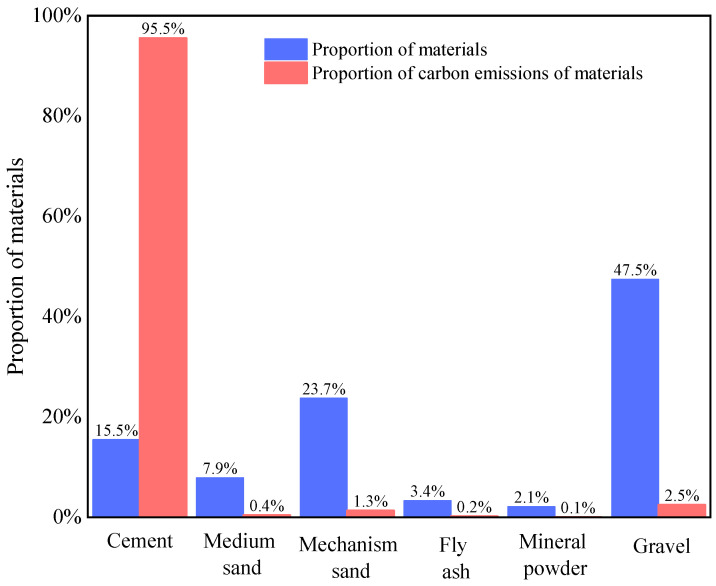
Proportions of the main materials and carbon emissions of C50 lightweight expanded concrete.

As shown in Figure 3, further analysis of materials with relatively high carbon emissions reveals that cement constitutes approximately 14% of 1 m^3^ of concrete, whereas other materials constitute approximately 86%. During production, cement is responsible for 95.5% of the total carbon emissions, whereas gravel and other materials account for only 4.5%. Thus, the majority of carbon emissions generated by high-energy-consuming concrete materials primarily originate from cement.

### 3.2. Carbon Emissions from the PCSPCs in the Production Phase

**Figure 4:**
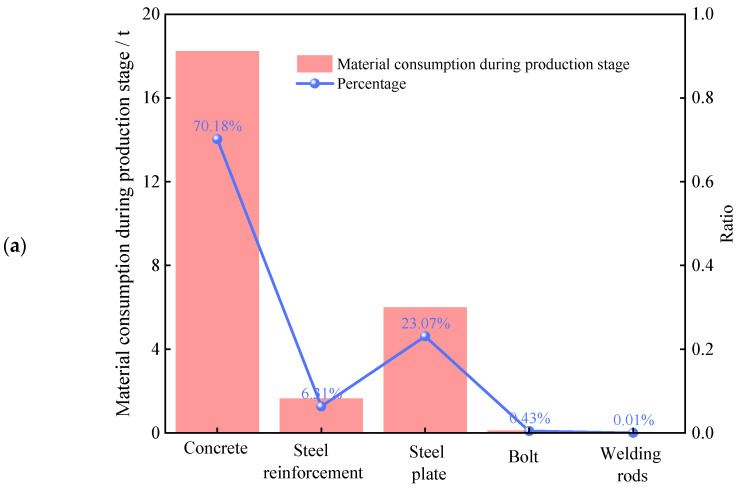

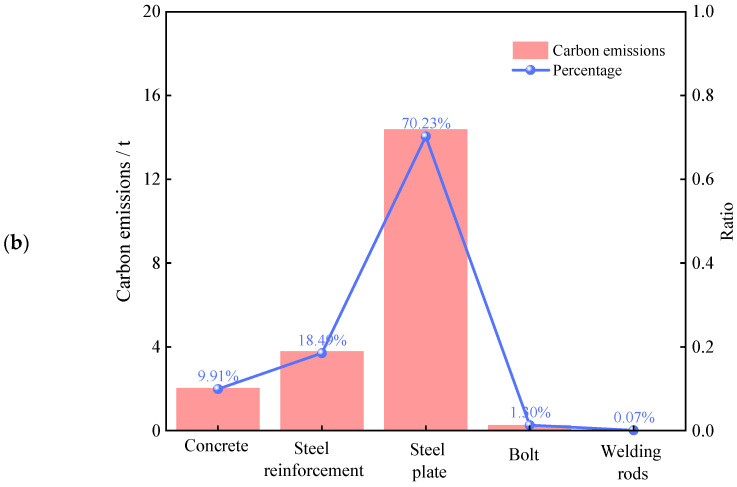
Material consumption at the production stage and its carbon emissions: (**a**) Material consumption. (**b**) Carbon emissions.

During the production phase, carbon emissions primarily arise from the use of building materials. As shown in Figure 4a, concrete accounts for more than 70% of the total material usage, steel plates approximately 24%, reinforcing bars approximately 7%, and bolts and welding rods less than 1%. However, Figure 4b reveals that steel plates contribute more than 70% of the carbon emissions, whereas reinforcing bars and concrete account for approximately 19% and 10%, respectively. Although the quantity of steel used is significantly lower than that of concrete, its carbon emissions are nearly twice as high. Similarly, while the amount of steel plate used is only one-third that of concrete, its carbon emissions exceed those of concrete by more than tenfold. This substantial disparity is due mainly to the much higher carbon emission coefficients of steel bars and steel plates than those of concrete, especially steel plates, whose complex smelting and processing involve substantial energy consumption, resulting in extremely high unit carbon emission intensity. The high carbon emission coefficient of steel stems primarily from its complex production chain. Starting with raw material extraction, iron ore mining typically involves large-scale excavation and long-distance transportation. The equipment used consumes significant energy and produces considerable carbon emissions. During smelting, high-carbon energy sources such as coal and coke are commonly employed to melt iron ore, releasing large volumes of carbon dioxide and other greenhouse gases. Furthermore, subsequent steel processing steps, such as rolling and forging, require substantial electricity or thermal energy, further increasing total carbon emissions. Consequently, steel plates and reinforcing bars generally exhibit high carbon emission coefficients.

### 3.3. Calculating the Material, Labor, and Construction Costs for PCSPCs

The total cost of PCSPCs can be categorized into material costs and construction-related costs, similar to how the carbon calculation phase is divided. The cost breakdown includes material costs, labor costs, and equipment costs, as shown in Equation (7). The material cost is calculated by the product of the material usage and the material unit price, which is referenced as the “Beijing Construction Project Pricing Basis”, as shown in Equation (8).

(7) $$Cost=\sum_{i}M_{Ci}+\sum_{i}\sum_{j}CC_{i,j}+M_{r}\times U_{cr}$$

where *M_Ci_* is the material cost for material; *CC_i,j_* is the equipment cost for using machinery *j* to process material *i*; *M_r_* is the total labor days; and *U_cr_* is the unit daily labor cost.

(8) $$M_{Ci}=U_{Ci}\times M_{i}$$

where *M_Ci_* is the material cost for material *i*; *U*_ci_ is the unit cost for material *i*; and *M_i_* is the quantity of material *i*.

Equipment costs cover all the machinery costs involved in transporting, lifting, and installing materials, whereas labor costs refer to the costs incurred in hiring labor. The above unit price is determined according to the Beijing urban rail transit project estimate quota. The final total cost calculation results are shown in Table 4, and the corresponding carbon emission results are shown in Table 5.

According to Table 4 and Table 5, the material cost dominates the total cost at 89.083%, whereas the construction machinery and labor costs account for 0.904% and 10.013%, respectively. Compared with the distribution of carbon emissions, the carbon emissions from construction machinery and labor accounted for 1.042% and 0.110% of the total carbon emissions, respectively.

**Figure 5:**
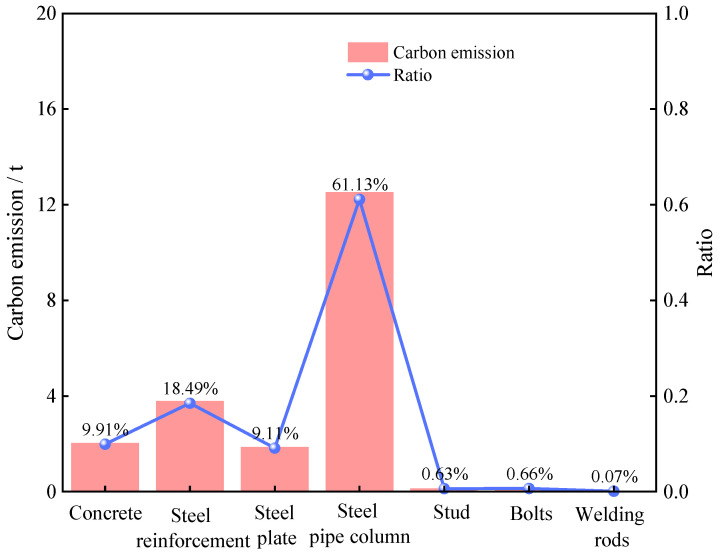
Material carbon emissions and proportions.

**Figure 6:**
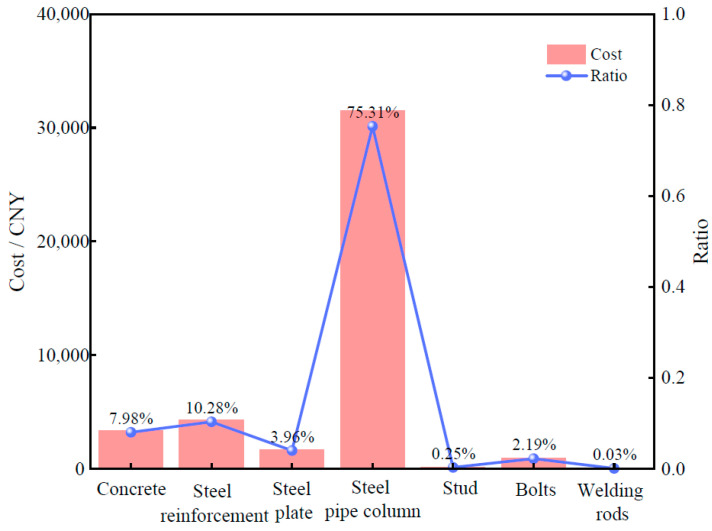
Material costs and proportions.

**Figure 7:**
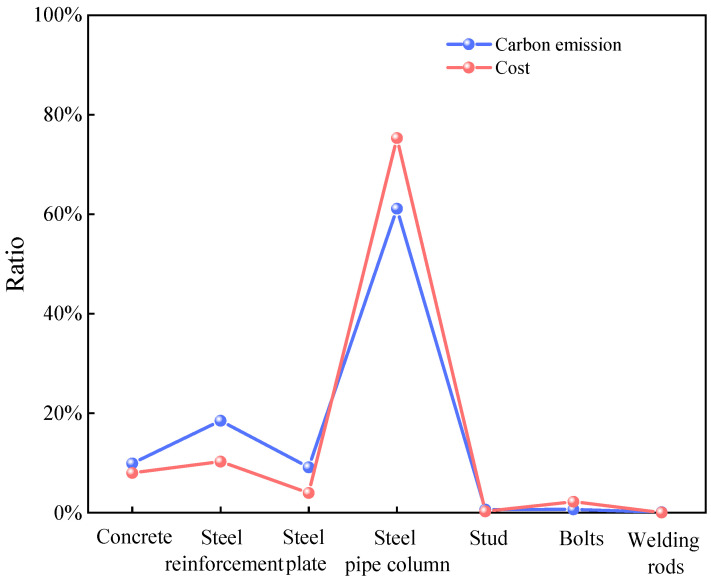
Material costs and carbon emissions.

To visualize the differences in different materials, we show the distributions of the cost and carbon emission of each major material in Figure 5. Figure 6 further visualizes the comparison between the carbon emissions of materials and the proportion of cost. Finally, Figure 7 synthesizes the correlation between the cost and the carbon emission to reveal the characteristics of the distribution of the two and their interrelationships in different materials.

An analysis of the results from Figure 5, Figure 6 and Figure 7 reveals that PCSPCs account for the largest portion of total material costs—approximately 75%—and that their carbon emissions constitute 62% of total material carbon emissions. This finding indicates that PCSPCs not only are dominant in cost but also significantly contribute to carbon emissions during production and use. Moreover, carbon emissions from steel reinforcement represent approximately 20% of total material emissions but only 10% of material costs, suggesting a relatively low cost–benefit ratio in the production and use of steel reinforcement, with carbon emissions disproportionately higher than its cost share. A comprehensive analysis considering both overall costs and carbon emissions reveals that the share of carbon emissions from manual operations is much lower than their cost share, whereas the difference between carbon emissions and the cost share from mechanized operations is smaller. This disparity indicates that manual operations in material production may incur higher costs but are less carbon-efficient, whereas mechanized operations, although they increase initial investment costs, are relatively more efficient in controlling carbon emissions and have a cost-to-emission balance that is more aligned.

**Figure 8:**
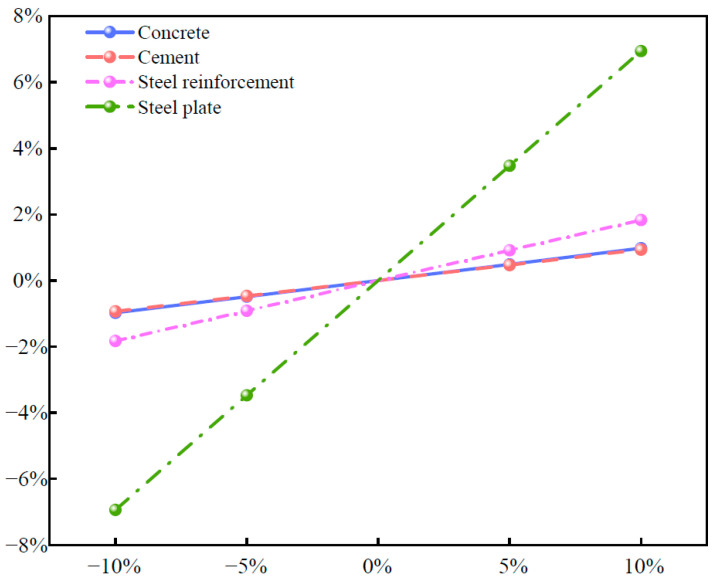
Sensitivity analysis of the concrete, cement, steel reinforcement, and steel plate.

During the construction process, accurately determining the workload is challenging because of high investment, complex procedures, and substantial labor requirements. Therefore, it is crucial to identify factors that have a greater impact on carbon emissions through sensitivity analysis and to propose targeted emission reduction measures. For PCSPCs, carbon emissions from the material production phase account for more than 90% of the total construction emissions. To evaluate the impact of energy efficiency technologies more comprehensively and clearly, different control scenarios were established, including a 5% reduction, a 5% increase, and a 10% increase in impact, as illustrated in Figure 8.

As shown in Figure 8, a 10% reduction in the carbon emission factor of steel plates results in an approximately 7% decrease in overall carbon emissions, whereas a 10% reduction in the emission factor of steel reinforcement leads to an approximately 3% decrease. A 10% reduction in cement emissions lowers total carbon emissions by less than 1%, with concrete showing a similar effect to cement, indicating that concrete’s carbon emissions primarily originate from cement. These findings demonstrate that, among the three control groups, steel plates exhibit the greatest change in carbon emissions, followed by steel reinforcement, with cement and concrete showing the smallest changes regardless of the magnitude of reduction. Although cement accounts for the largest share of carbon emissions within concrete, it has relatively low sensitivity to overall carbon emissions, whereas steel plates display high sensitivity. Therefore, when selecting construction materials, emission reduction strategies should be developed according to the carbon emission sensitivity of each material, prioritizing green and environmentally friendly options to support energy savings and emission reductions in urban underground rail transit construction.

## 4. Optimization of the Carbon Emission and Cost of Steel Pipe Columns via an Iterative Algorithm

### 4.1. Ultimate Bearing Capacity Calculation of the PCSPCs

Several methods have been developed for predicting the load-carrying capacity of steel-tube concrete members, but most of them are only applicable when the design variables, such as the yield strength of steel and the compressive strength of concrete, are within a specific range. In this paper, on the basis of the Technical Specification for Steel Pipe Concrete Structures published by the Ministry of Housing and Urban-Rural Development of the People’s Republic of China, the corresponding formulas are derived, as shown in Equation (9):

(9) $$\left\{\begin{array}{l} N_{u}=\varphi_{l}\times N_{0}\\ N_{0}=0.9\times A_{c}\times f_{c}\times \left(1+\alpha \times \theta \right)\\ \theta =A_{s}\times \frac{f}{A_{c}}\times f_{c}\\ \varphi_{l}=1-0.0226\times \left(\frac{L_{e}}{D}-4\right)\\ L_{e}=\mu \times k\times L \end{array}\right.$$

where *N_u_* is the ultimate bearing capacity of the PCSPCs, *N*_0_ is the design value of the strength-bearing capacity of the PCSPCs axial compression, *θ* is the hoop coefficient of the PCSPCs members, *α* is the coefficient related to the strength grade of the concrete, *A_c_* is the cross-sectional area of the core concrete in the steel pipe (mm^2^), *f_c_* is the design value of the compressive strength of the core concrete in the steel pipe (MPa), and *A_s_* is the cross-sectional area of the steel pipe (mm^2^). *f* is the design value of the tensile and compressive strengths of steel pipes (MPa), *φ_l_* is the bearing capacity reduction factor considering the influence of the length and slenderness ratio, *D* is the outer diameter of the PCSPCs (mm), *t* is the wall thickness of the PCSPCs, *L_e_* is the equivalent calculated length of the PCSPCs (mm), *L* is the actual length of the PCSPCs (mm), *μ* is the coefficient of the calculated length considering the constraint condition of the column end, which should be executed according to the current national standard “Code for the Design of Steel Structures” GB-50017 [35], and *k* is the equivalent length coefficient considering the effect of the gradient of the bending moment distribution of the column body.

### 4.2. Iterative Algorithm Optimization of the PCSPCs

To further evaluate the optimal combination of PCSPCs’ ultimate bearing capacity, carbon emissions, and costs, we employed a hierarchical grid search algorithm based on deterministic parameter space scanning and iterative refinement. The primary objective is to minimize both carbon emissions and costs while satisfying the ultimate bearing capacity constraint. Specifically, the algorithm first constructs an initial parameter space by discretizing the steel pipe column diameter, wall thickness, and concrete strength grade within a reasonable engineering range, forming an initial three-dimensional parameter grid matrix. For each parameter combination, the corresponding ultimate bearing capacity, carbon emissions, and construction costs are calculated, and feasible solutions meeting the minimum bearing capacity requirements are selected. Within this feasible set, the algorithm identifies the parameter combination that minimizes carbon emissions or construction costs as the optimal solution for the current iteration. The variable step size is subsequently gradually reduced to create a higher-resolution grid around the optimal solution, and the process is subsequently repeated. When the difference between the optimal target values of two consecutive iterations falls below a predefined threshold, the process is considered converged, and the optimization terminates.

Based on the bearing capacity equation, carbon emissions were constrained and optimized under different bearing capacity conditions. In data processing and statistical analysis, parameter selection is a crucial factor in determining the frequency and quantity of samples extracted from the overall dataset. The initial selection is essentially a ratio chosen according to the research objectives, dataset size, and required accuracy. Once the initial selection is set, the parameter combination matrix can be constructed. This matrix records information about different parameter combinations, with each element representing the value of a variable. In cases with multiple variables, each item corresponds to the coordinate values sampled for each variable. The process of generating the sampling matrix can be summarized as follows: first, the required number of samples is determined on the basis of the initial sampling rate, which defines the dimensions of the sampling matrix. Next, samples are randomly or systematically drawn from the raw data according to the sampling rate, typically using a random number generator or a specific sampling algorithm to ensure representativeness. The specific values for each variable are then recorded for each extracted sample, forming the elemental coordinates of the sampling matrix. Finally, these coordinate values are populated into the sampling matrix, completing its structure. This matrix not only reflects the spatial distribution of sample points but also reveals potential relationships between variables and data distribution characteristics.

**Figure 9:**
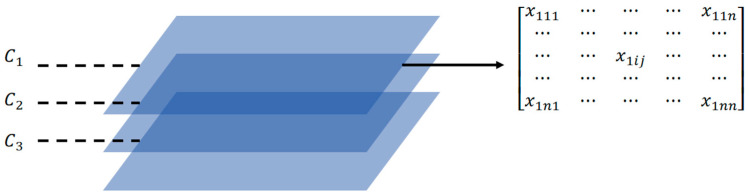
Sampling matrix.

The initial parameter discrete step size *n* is set, and a 3 × *n* × *n* parameter combination matrix is generated in the design variable space for multiobjective optimization calculations. The sampling matrix *X* = [*x*_kij_] is generated on the basis of parameter combinations, where the three coordinates of the matrix elements refer to the three variables *x*_1_, *x*_2_, and *x*_3_, as shown in Figure 9. In the figure, *C*_1_ represents the diameter parameter group of the steel pipe columns, *C*_2_ represents the wall thickness parameter group of the steel pipe columns, and *C*_3_ represents the concrete strength grade parameter group. These three parameter groups together constitute the three-dimensional design variable space used for traversal in multiobjective optimization.

The carbon emission *Y*_1*q*_ = [*y*_1*qkij*_] corresponds to all the sampling points, and the ultimate bearing capacity of the steel pipe column *Y*_2*q*_ = [*y*_2*qkij*_] can be obtained via matrix operation, where q represents the number of cycles. According to the load-bearing conditions in Equation (10), the *y*_1*qkij*_ that meets the requirements is screened out, and its minimum value min*y*_1*q*_ is found.

(10) $$y_{2qkij}<y_{1qkij}$$

To achieve a controlled parameter search, a grid discretization method was employed to traverse combinations of the steel pipe column diameter, wall thickness, and concrete strength grade. Owing to the discrete nature of these parameters, the optimal solution is subject to certain accuracy limitations. Therefore, the optimization process is iteratively repeated by gradually increasing the resolution of the parameter grid (i.e., refining the step size). When the difference between the optimal carbon emissions (or cost) obtained in two consecutive iterations falls below a predefined threshold, the algorithm is considered to have converged and met the accuracy requirements. The sampling rate *n* is then increased, and the process repeats until the difference between the results of two successive cycles is less than the specified error, as shown in Equation (11).

(11) $$\min y_{1q}<\min y_{1q-1}$$

**Figure 10:**
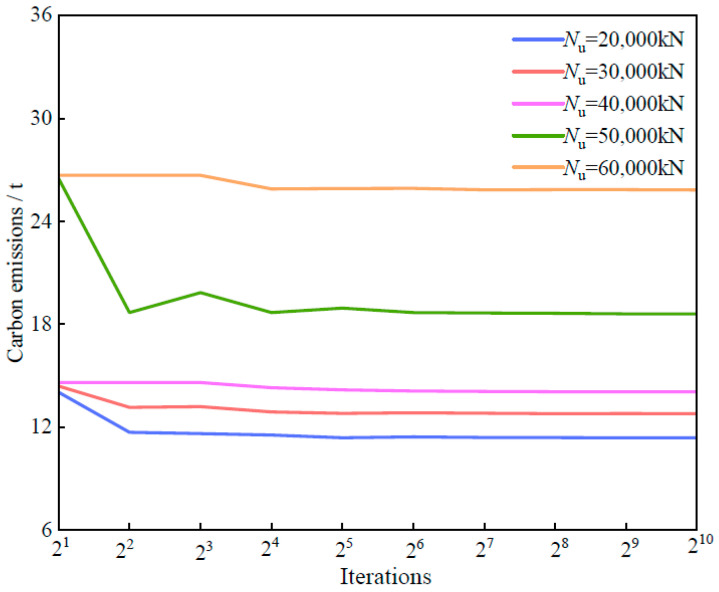
Carbon emission optimization under different bearing capacities.

The end loop outputs the results and outputs the corresponding coordinates, thus obtaining the lowest carbon emission and cost. The optimal carbon emissions for different carrying capacities were calculated through 210 iterations and are shown in Figure 10.

According to the optimization results shown in Figure 10, when the ultimate load capacity is 50,000 kN, with a component diameter of 1000 mm and a wall thickness of 16.53 mm, the optimal carbon emission is 18.59 t. Compared with the design with a maximum load capacity of 40,000 kN, the load capacity increases by 25%, whereas the carbon emission increases by approximately 30%. Although the increase in carbon emissions slightly exceeds the gain in load-bearing capacity, it remains within a manageable range overall, indicating that the optimization scheme effectively controls carbon emissions while enhancing structural performance.

**Figure 11:**
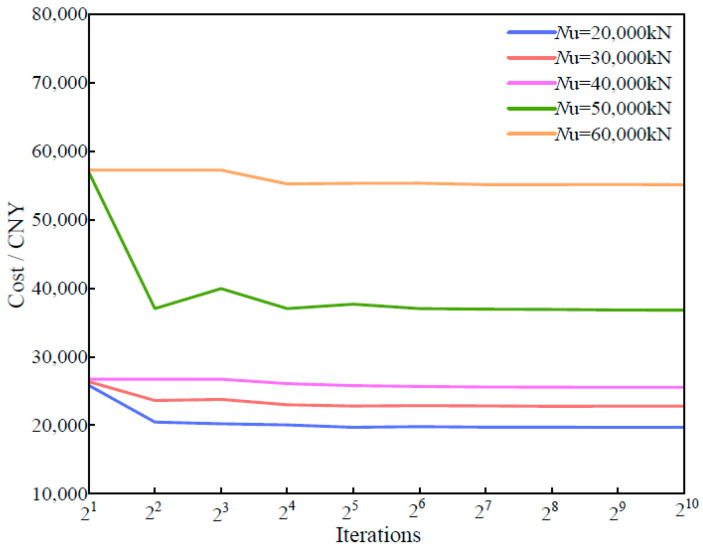
Cost optimization under different bearing capacities.

**Figure 12:**
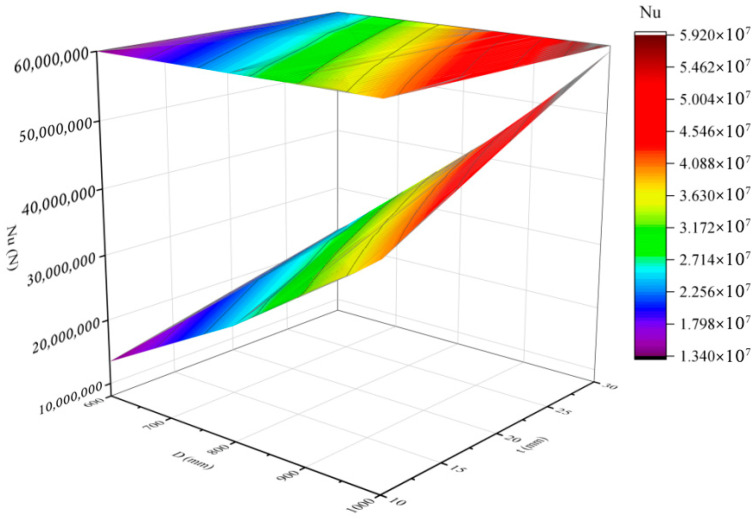
Ultimate load capacity varies with geometry.

In terms of cost optimization, the same multiobjective optimization algorithm was applied, with concrete grades limited to the C30–C60 range and load-bearing capacities set between 20,000 and 60,000 kN. The optimal results are shown in Figure 11. On the basis of the case analysis, when the ultimate load-bearing capacity reaches 50,000 kN, the lowest total cost is CNY 36,825.322. Compared with the solution with a 40,000 kN load-bearing capacity, the capacity increases by 25%, whereas the cost increases by approximately 44%. Although the cost increase is significant, this solution still offers high engineering value for structural designs requiring elevated load-bearing capacity and stringent safety standards. Moreover, when both costs and carbon emissions are optimized, the results across different load-bearing conditions display a consistent trend. This can be attributed to the dominant influence of material properties within the PCSPCs structure. Specifically, the carbon emission coefficient and unit cost of steel plates are roughly ten times greater than those of concrete. Consequently, under various load-bearing capacities, steel plates consistently have the greatest impact on total carbon emissions and overall cost. This dominance results in similar optimization trends regardless of the objective function. Figure 12 illustrates the trend of load-bearing capacity as a function of cross-sectional dimensions—particularly steel plate thickness—further confirming the central role of steel plates in the optimization process.

### 4.3. Verification of the Optimization Results

To verify the influence of the optimal solution obtained through the iterative algorithm on the structural safety of PCSPCs at the overall station, simplified modeling of several standard sections of the subway station—constructed via the PBA method—was performed via finite element analysis software ABAQUS 6.1. The design parameters of the PCSPCs were adjusted within the model to conduct a comparative analysis before and after optimization. The validity and reliability of the optimization scheme were further confirmed by comparing the structural load-bearing capacity of the metro station under the PBA method before and after PCSPCs optimization. Numerical software was employed to model and analyze the structural performance of steel-tube–concrete columns, with a focus on the interaction between the steel tube and concrete, load application, and boundary condition settings.

First, a three-dimensional model of the steel pipe and concrete was created, where the steel pipe was formed by extruding a two-dimensional circular cross-section into a cylindrical shape, and the concrete was modeled as a solid cylinder on the basis of the internal cross-sectional dimensions of the steel pipe. For material properties, the density, elastic modulus, and strength of the concrete were assigned according to the Concrete Design Code, with an appropriate dilation angle selected in the plastic damage model to ensure convergence of the calculation. The steel pipe material properties, including density and Poisson’s ratio, were assigned to uniform solid cross-sections. In terms of assembly and interaction, the contact between the steel pipe and concrete was defined via a hard contact model in the normal direction to simulate the physical interaction accurately between the inner wall of the steel pipe and the concrete. The contact surface remains constrained under normal pressure but allows separation under tensile stress, reflecting actual cracking or detachment phenomena. A penalty friction model based on Coulomb friction theory with a friction coefficient of 0.3 was subsequently applied to represent shear force transfer caused by finite slip, accurately capturing the slip mechanism at the steel–concrete interface. A fully fixed constraint was applied to the bottom surface of the column to simulate a rigid connection with the foundation. Axial displacement-controlled loads were applied to the top of the column via a coupled reference point, effectively preventing numerical instability caused by load concentration. This reference point was fully coupled to all nodes at the top surface through multipoint constraints, ensuring even load distribution. Both the steel pipe and the concrete were meshed via eight-node linear hexahedral reduced integration elements (C3D8R) to increase the convergence and computational efficiency. The steel pipe wall thickness was modeled with at least two layers to better capture the local yielding and bending behavior. The overall mesh size was determined through convergence analysis, with moderate mesh refinement applied in the concrete core area.

**Figure 13:**
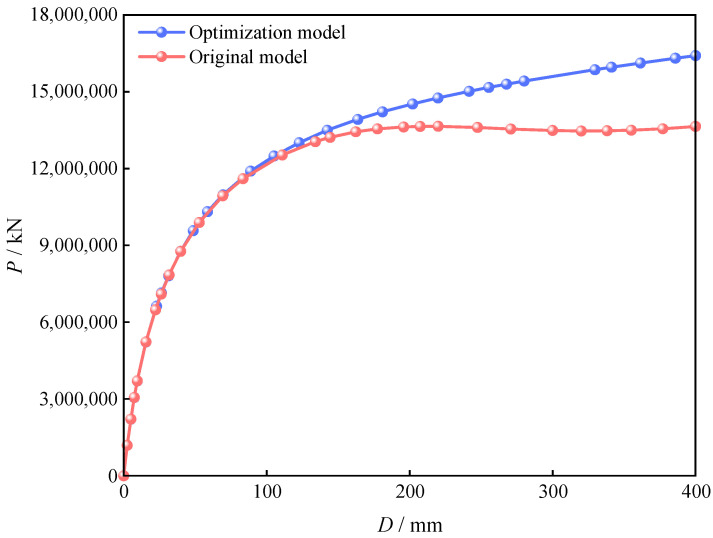
Load–displacement curves of the PBA station before and after optimization of the PCSPCs.

To assess the ultimate bearing capacity of the metro station structure more accurately, displacement loads were applied at the top during numerical simulation, with the resulting load–displacement curves shown in Figure 13. The results indicate that the overall load-bearing capacity of metro stations constructed via the PBA method combined with the optimized PCSPCs strategy is significantly greater than that of stations built via the preoptimization model. Under the same displacement conditions, the optimized structure remains within the elastic stage, whereas the unoptimized model has already entered the yielding phase, clearly demonstrating the advantage of the optimized design in delaying structural failure. Furthermore, the results show that the optimization scheme not only improves load-bearing performance but also effectively reduces material consumption and carbon emissions while controlling construction costs, further validating the practical applicability and engineering feasibility of the PBA optimization strategy in metro construction.

**Figure 14:**
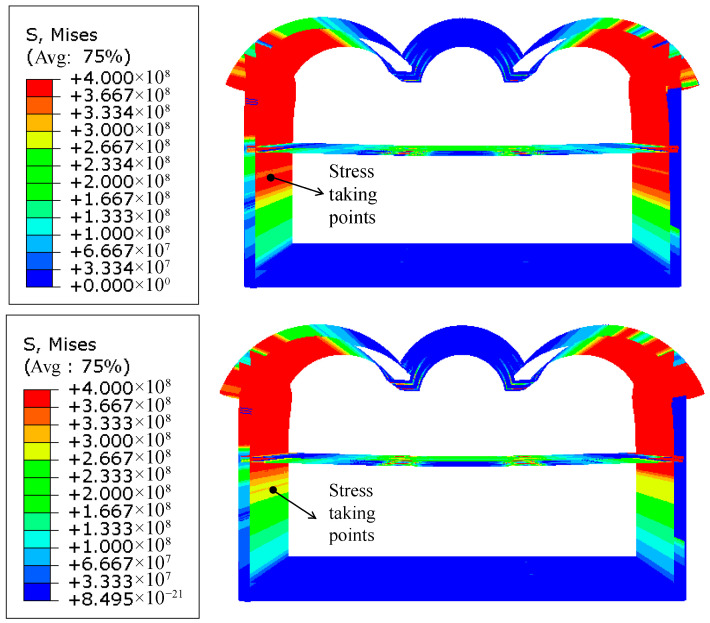
Stress clouds of the stressed steel bars in the station structure before and after optimization of the PCSPCs.

To further evaluate the effectiveness of the PBA-optimized steel column structure in enhancing the load-bearing capacity of metro stations, models before and after optimization were compared with actual stress conditions observed in engineering cases, as shown in Figure 14. The results indicate that, under identical displacement conditions, stress development in the bottom wall reinforcement of the optimized model progresses more gradually, resulting in delayed yielding, whereas the reinforcement in the unoptimized model reaches its yield limit earlier, highlighting a significant difference in structural load-bearing performance.

**Figure 15:**
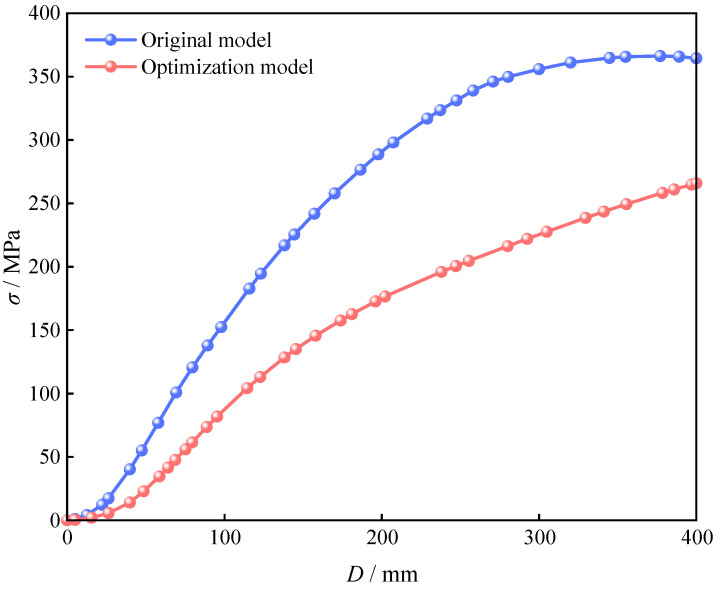
Stress–displacement curves of force reinforcement in the ground floor of the station before and after optimization of the PCSPCs.

As shown in Figure 15, the stress–displacement curve of the reinforcing bars indicates that, under the same displacement conditions, the reinforcing bars in the bottom wall of the original metro station model have reached the yield state, whereas those in the optimized PCSPCS model remain in the elastic stage. These results demonstrate that the optimized design not only delays the yielding of the reinforcing bars and enhances the load-bearing capacity of the wall but also improves the overall structural performance and reduces carbon emissions by optimizing the material distribution and stress paths.

To verify the reliability of the numerical calculations, the simulation results are compared with the calculation results in this paper, as shown in Table 6, Table 7 and Table 8.

As shown in Table 6, Table 7 and Table 8, when the diameter of the steel pipe column is 1000 mm, the calculated and simulated values are highly consistent, with significantly reduced errors. However, as the concrete strength grade increases, the gap between the calculated and simulated values gradually widens, particularly at the C60 grade, where the error is noticeably greater than those at C30 and C50. Further analysis of the simulation and calculation results for different diameters reveals that larger diameters correspond to smaller errors. Specifically, when the diameter is 1000 mm, a relatively high proportion of the errors are controlled within 1000 kN, indicating that the diameter significantly affects the accuracy of the results. Under fixed concrete strength conditions, the ultimate bearing capacity of steel pipe columns increases with increasing diameter or wall thickness, with the diameter having a particularly significant effect. In contrast, for components with a diameter of 600 mm, increasing the wall thickness leads to only slight improvements in the load-bearing capacity, indicating that simply increasing the wall thickness is not the optimal approach for enhancing the capacity. These results indicate that the ultimate bearing capacity generally increases with increasing component diameter, wall thickness, and concrete strength grade. Under the same concrete grade and wall thickness, increasing the diameter from 600 mm to 800 mm and 1000 mm significantly enhances the load-bearing capacity, demonstrating the dominant role of cross-sectional dimensions in component performance. At a fixed diameter, increasing the wall thickness also substantially improves the load-bearing capacity, especially with high-strength concrete such as C50 and C60. Overall, higher concrete strength corresponds to greater ultimate load-bearing capacity, with C60 components exhibiting significantly higher capacity than C30 and C50 under identical geometric parameters.

### 4.4. Discussions

In the carbon emission modeling process of this study, to ensure clarity in the analytical framework and operability of the calculations, some parameters were based on typical engineering assumptions or empirical settings. These assumptions primarily include averaging transportation distances, using actual onsite consumption data for building materials, selecting carbon emission factors from publicly available databases, and excluding carbon emissions from nonprimary processes such as construction waste removal and earthworks. Although sensitivity analysis was not conducted for each data point, these assumptions are reasonable within the scope of the current study. First, transportation distances were determined through communication with the project unit and onsite surveys, reflecting typical routing arrangements for most materials from local procurement to the construction site. While this assumption does not account for extreme cases, it is common in most urban subway projects and is therefore representative. Second, material usage data were obtained from actual construction records, which better reflect real carbon emissions than theoretical quantities from design drawings. This approach also naturally accounts for construction losses, providing a robust basis for carbon emission assessment. Furthermore, carbon emission factors were selected on the basis of standardized databases and local statistical data. Although differences in material sources and processing methods cannot be fully covered, this practice is widely accepted in engineering carbon assessments. Given that this study aims to evaluate the relative carbon efficiency of different design schemes rather than absolute precise values, this approach is methodologically sound. Finally, certain carbon emissions—such as those from construction waste, nonstructural materials, and temporary facilities—were excluded on the basis of their relatively small proportion of total construction emissions and their limited direct impact on the optimization of the main structural design. This simplification allows a focus on key factors with significant impact. However, these assumptions may have limitations in different project contexts. Future research could adopt more refined carbon emission process modeling methods, gradually incorporating additional influencing factors to improve assessment comprehensiveness and model generality. In summary, the following points are discussed:

(1) A high proportion of carbon emissions in the production stage and research significance: This study shows that, in the full life cycle of PCSPCs for metro stations, the production stage accounts for more than 90% of total carbon emissions, with the material production process alone contributing as much as 98.9%. In contrast, the transportation and construction stages contribute only 0.3% and 0.8%, respectively. Compared with aboveground buildings, the use of the PBA method in metro station construction results in a greater proportion of emissions from the production stage. Therefore, an in-depth investigation of the production stage—particularly material manufacturing—is crucial for reducing future carbon emissions in underground structures, optimizing design, and mitigating environmental impacts.
(2) Sensitivity of high-carbon materials and emission reduction priorities: A sensitivity analysis of four major construction materials with high carbon emissions during production revealed that steel plates have the highest sensitivity (>6%), followed by steel reinforcement (<2%), whereas cement and concrete have similar sensitivities (both <1%). This indicates that emission reduction strategies should prioritize greenhouse gas-intensive materials, especially steel plates and steel reinforcements. The recommended measures include selecting environmentally friendly alternatives, increasing the recycling rates of steel structures, and adopting processes that reduce the carbon footprint associated with material use.
(3) Strategies for material substitution and design optimization: For high-carbon materials such as steel plates and steel reinforcements, promising alternatives such as fiber-reinforced composites (e.g., CFRPs) can significantly reduce carbon emissions due to their excellent strength–weight ratio, which lowers transportation energy use and structural dead weight. Furthermore, advanced topology optimization can refine the layout and cross-section of steel elements on the basis of the actual stress distribution, avoiding material overuse. Coupled with efficient recycling and reuse systems—where recovered materials are applied to noncritical components—these measures can extend material life cycles and substantially reduce emissions from new material production.

## 5. Conclusions

(1) This study employed the LCA method to develop a carbon emission calculation model for the construction phase of PCSPC structures at PBA subway stations and established a carbon emission factor database. The results show that carbon emissions during the material production stage account for more than 98% of total emissions, with steel plates contributing the most (>70%), followed by steel bars (approximately 19%) and concrete (approximately 10%). Carbon emissions from the transportation and construction stages account for less than 1.2% of total emissions.
(2) Cost analysis reveals that material costs account for 88.72% of total costs, which is consistent with their share of carbon emissions (98.85%). Although labor accounts for 10.34% of costs, it contributes only 0.11% of carbon emissions. This finding indicates that materials are the primary source of both costs and carbon emissions and that energy conservation and emission reduction efforts should focus on the material stage.
(3) By combining load-bearing capacity constraints, structural parameter optimization was achieved through an iterative algorithm. The optimized PCSPC structure reduced carbon emissions by 10.32% and costs by 21.55% while still meeting load-bearing capacity requirements. Finite element analysis further verified the effectiveness and feasibility of the optimization scheme in improving the structural load-bearing performance.
(4) The carbon emission calculations in this study rely on existing carbon emission factor databases, which may be subject to geographical limitations and data update delays. The actual energy consumption and carbon emissions during the construction phase cannot be dynamically monitored, which affects the timeliness and accuracy of the model. Additionally, structural optimization considers relatively simple load conditions and does not fully account for complex construction environments or multiobjective optimization requirements. In the future, the integration of BIM technology, intelligent construction monitoring, and low-carbon material databases can facilitate the development of a multidimensional carbon emission prediction and control platform, enabling dynamic carbon management from the design stage through the entire construction process. Moreover, research on alternative materials and structural forms should be strengthened to promote the deep integration of PBA-method subway stations with green construction, intelligent optimization, and sustainable development.

## Figures and Tables

**Table 1:** Building material carbon emission factors.

Types of Materials	Carbon Emission Factors	Unit
Steel reinforcement	2.309	tCO_2_/t
Steel plate	2.4	tCO_2_/t
Cement	0.735	tCO_2_/t
Mechanism sand	0.0066	tCO_2_/t
C50 lightweight expanded concrete	0.385	tCO_2_/m^3^
Fly ash	0.008	tCO_2_/t
Mineral powder	0.003	tCO_2_/t
Steel	1.959	tCO_2_/t
Fine sand	0.00251	tCO_2_/t
Medium (coarse) sand	0.004	tCO_2_/t
Crushed stone (1~3 cm)	0.00218	tCO_2_/t
Crushed stone (4 cm)	0.003	tCO_2_/t
Mortar	0.004	tCO_2_/t
Bolt	2.35	tCO_2_/t
Welding rods	9.53	tCO_2_/t

**Table 2:** Carbon emissions from transportation.

Materials	Total Quantity (t)	Carbon Emission Factors (kgco_2_/t·km)	Total Carbon Emissions
Steel reinforcement	1.641	0.104	0.806 kg
Steel plate	5.997	0.104	
Bolt	0.113	0.104	

**Table 3:** Estimation of total carbon emissions in the construction stage of the PCSPCs.

Materials	C50 lightweight expanded concrete	m^3^	7.813	0.260	2.031
Steel plate	Steel reinforcement	t	1.641	2.309	3.789
Bolt	Steel plate	t	5.997	2.4	14.393
Materials	Bolts	t	0.113	2.35	0.266
	Welding rods	t	0.00158	9.53	0.015
Construction machinery	AC welding machine	Shift	1.80	0.074	0.133
	Crane	Shift	0.4186	0.167	0.070
	Concrete pump truck	Shift	0.3591	0.035	0.0126
Artificial	Total man-days	Man-day	49.75	0.00046	0.0229
Total					20.733

**Table 4:** Materials, construction machinery, and labor costs for PCSPCs.

Types	Unit	Quantity	Unit Cost	Cost	Total Cost	Percentage
Materials	C50 lightweight expanded concrete	m^3^	7.813	427.48	3339.901	89.083%
	Steel reinforcement	t	1.641	2620	4299.42	
	Steel plate	t	0.777	2130	1655.01	
	20 mm thick PCSPCs	t	5.22	6034.19	31,498.472	
	Stud	Pcs	64	1.64	104.96	
	Bolts	Set	60	15.3	918	
	Welding rods	t	0.00158	6650	10.507	
Construction machinery	AC welding machine	Shift	1.80	15.38	27.684	0.904%
	Crane	Shift	0.4186	585.83	245.228	
	Concrete pump truck	Shift	0.3591	422.56	151.741	
Artificial	Total man-days	Man-day	49.75	94.5	4701.375	10.013%
Total				46,941.791 (100%)		

**Table 5:** The cost and carbon emissions of materials, construction machinery, and labor.

Types	Cost (¥)	Percentage	Total Carbon Emissions (t)
Materials	41,826.270	89.083%	20.494
Construction machinery	424.653	0.904%	0.216
Artificial	4701.375	10.013%	0.0229

**Table 6:** Comparative simulation results for D = 1000 mm.

Serial No.	Diameter (mm)	Thickness (mm)	Concrete Grade	Calculated Value (kN)	Simulated Value (kN)
1	1000	10	C30	28,370.38	25,988.8
2	1000	20	C30	38,583.34	39,098.8
3	1000	30	C30	48,585.71	48,426.2
4	1000	10	C50	39,861.15	39,892.3
5	1000	20	C50	49,609.88	52,041.4
6	1000	30	C50	59,157.61	60,110.5
7	1000	10	C60	44,493.11	49,440.4
8	1000	20	C60	52,918.80	59,839.8
9	1000	30	C60	61,170.76	66,362.2

**Table 7:** Comparative simulation results for D = 800 mm.

Serial No.	Diameter (mm)	Thickness (mm)	Concrete Grade	Calculated Value (kN)	Simulated Value (kN)
1	800	10	C30	18,375.89	21,683.7
2	800	20	C30	25,898.55	28,753
3	800	30	C30	33,225.82	33,921.9
4	800	10	C50	25,130.30	30,493.9
5	800	20	C50	32,311.02	36,294.9
6	800	30	C50	39,305.23	39,824.4
7	800	10	C60	27,683.07	31,783.3
8	800	20	C60	33,889.27	40,692.4
9	800	30	C60	39,934.26	44,077.6

**Table 8:** Comparative simulation results for D = 600 mm.

Serial No.	Diameter (mm)	Thickness (mm)	Concrete Grade	Calculated Value (kN)	Simulated Value (kN)
1	600	10	C30	10,236.29	11,536.9
2	600	20	C30	15,083.83	17,766.5
3	600	30	C30	19,761.29	21,957.6
4	600	10	C50	13,487.31	16,513.9
5	600	20	C50	18,114.52	21,214
6	600	30	C50	22,579.36	24,744.6
7	600	10	C60	14,576.85	18,059.1
8	600	20	C60	18,576.08	20,578.4
9	600	30	C60	22,434.98	27,085.4

## Data Availability

The original contributions presented in this study are included in the article. Further inquiries can be directed to the corresponding authors.
